# Association and Interaction between Heavy Metals and Hyperuricemia in a Taiwanese Population

**DOI:** 10.3390/diagnostics13101741

**Published:** 2023-05-15

**Authors:** Lu-Heng Lu, Chun-Chi Tsai, Chih-Yi Lin, Chih-Wen Wang, Pei-Yu Wu, Jiun-Chi Huang, Szu-Chia Chen, Jer-Ming Chang

**Affiliations:** 1 Department of General Medicine, Kaohsiung Medical University Hospital, Kaohsiung Medical University, Kaohsiung 807, Taiwan; luke01099314199@gmail.com; 2 Department of Occupational Safety and Health, Kaohsiung Municipal Siaogang Hospital, Kaohsiung Medical University, Kaohsiung 812, Taiwan; 1036124@kmuh.org.tw; 3 Department of Environmental and Occupational Medical Center, Kaohsiung Municipal Siaogang Hospital, Kaohsiung Medical University, Kaohsiung 812, Taiwan; joannalinnn0824@gmail.com (C.-Y.L.); chinwin.wang@gmail.com (C.-W.W.); 4 Department of Internal Medicine, Kaohsiung Municipal Siaogang Hospital, Kaohsiung Medical University Hospital, Kaohsiung Medical University, Kaohsiung 812, Taiwan; wpuw17@gmail.com (P.-Y.W.); karajan77@gmail.com (J.-C.H.); 5 Division of Hepatobiliary, Department of Internal Medicine, Kaohsiung Medical University Hospital, Kaohsiung Medical University, Kaohsiung 807, Taiwan; 6 Faculty of Medicine, College of Medicine, Kaohsiung Medical University, Kaohsiung 807, Taiwan; 7 Division of Nephrology, Department of Internal Medicine, Kaohsiung Medical University Hospital, Kaohsiung Medical University, Kaohsiung 807, Taiwan; 8 Research Center for Precision Environmental Medicine, Kaohsiung Medical University, Kaohsiung 807, Taiwan

**Keywords:** heavy metal, arsenic, hyperuricemia, risk factors

## Abstract

The prevalence of hyperuricemia in Taiwan is high, and hyperuricemia has been associated with a risk of developing several diseases. Although the traditional risk factors for hyperuricemia are well known, the relationship between heavy metals and hyperuricemia is still undefined. Therefore, the aim of this study was to investigate the relationship between hyperuricemia and heavy metals. A total of 2447 participants (977 males and 1470 females) residing in southern Taiwan were enrolled, and levels of the following heavy metals were measured: lead in blood, and nickel, chromium, manganese, arsenic (As), copper, and cadmium in urine. Hyperuricemia was defined as a serum uric acid level greater than 7.0 mg/dL (416.5 μmol/L) in men and 6.0 mg/dL (357 μmol/L) in women. The participants were divided into two groups: those without hyperuricemia (*n* = 1821; 74.4%) and those with hyperuricemia (*n* = 626; 25.6%). Multivariate analysis showed that only high urine As (log per 1 μg/g creatinine; odds ratio, 1.965; 95% confidence interval, 1.449 to 2.664; *p* < 0.001), young age, male sex, high body mass index, high hemoglobin, high triglycerides, and low estimated glomerular filtration rate were significantly associated with hyperuricemia. In addition, the interactions between Pb × Cd (*p* = 0.010), Ni × Cu (*p* = 0.002), and Cr × Cd (*p* = 0.001) on hyperuricemia were statistically significant. Increasing levels of Pb and Cr yielded an increased prevalence of hyperuricemia, and the effect was progressively greater for increasing Cd. Moreover, increasing levels of Ni yielded an increased prevalence of hyperuricemia, and the effect was progressively greater for increasing Cu. In conclusion, our results show that high urine As is associated with hyperuricemia, and some interactions of heavy metals on hyperuricemia are noted. We also found that young age, male sex, high BMI, high hemoglobin, high triglycerides, and low eGFR were significantly associated with hyperuricemia.

## 1. Introduction

The prevalence of hyperuricemia in Taiwan is higher compared with other countries [1]. According to data from Nutrition and Health Surveys conducted in Taiwan from 2005–2008, the prevalence of hyperuricemia was 22.0% in men and 9.7% in women when hyperuricemia was defined as a serum uric acid level ≥ 7.7 mg/dL (458.5 μmol/L) in men and ≥6.6 mg/dL (392.7 μmol/L) in women or the use of uric acid-lowering drugs [2]. Uric acid is the end product of purine metabolism in humans [3], and it is produced by xanthine oxidase catalyzing hypoxanthine and xanthine [4]. Reactive oxygen species are generated during this process, and they have been shown to contribute to metabolic dysfunction [3]. These mechanisms may imply a causal role of hyperuricemia in several systemic diseases. Known risk factors for hyperuricemia include old age, male sex, obesity, and poor renal function [4,5,6]. Hyperuricemia is not only associated with an increased risk of gout [4], but also cardiovascular abnormalities such as a high left atrial diameter and low left ventricular ejection fraction [7]. Hyperuricemia is also a potential risk factor for chronic kidney disease (CKD), metabolic syndrome, hypertension, dyslipidemia, and thyroid dysfunction [4,6,7,8]. Hence, identifying contributors to hyperuricemia is important to further decrease the burden of disease on society.

Anthropogenic activities create various problems such as greenhouse gas emissions, ultraviolet radiation, water pollution, air pollution, and heavy metal exposure [9]. The use of heavy metals has mostly been accompanied with the development of industrialization and urbanization, and has resulted in an environmental crisis. Some metals, such as manganese (Mn), zinc, copper (Cu), and iron, are essential for the human body [10], but have toxic effects if ingested at higher concentrations. Other heavy metals, such as mercury and lead (Pb), have no benefit to our health and are considered to be harmful [10]. Due to the increase in industrialization, the deleterious effects of heavy metals on human health is a growing concern worldwide. A study from Wuhan City [11], Central China, analyzed heavy metals in street dust and found that they mainly originated from industrial processes and traffic pollution. Street dust enters the body through the ingestion of polluted food or drinking water, skin contact and respiratory inhalation. Once heavy metals enter the body they can accumulate in vital organs such as the liver, heart, kidneys, and brain, inducing the generation of reactive oxygen species and subsequently oxidative stress [12]. These effects ultimately disturb normal biological functioning leading to an internal imbalance [12]. Several studies have demonstrated that heavy metals are critical risk factors for neurologic diseases, metabolic abnormalities, cardiovascular diseases, kidney damage, and various types of cancer [12,13,14,15,16,17].

A study from the United States evaluated the effect of arsenic (As) exposure on uric acid, and found that high As exposure was associated with an increased prevalence of hyperuricemia in men [13]. However, another study conducted in eastern India revealed a reverse relationship between high As levels in drinking water and uric acid level [14]. Despite the plausible relationship between exposure to heavy metals and the risk of hyperuricemia, previous studies have reported inconsistent findings. The additive, synergistic, or antagonistic effects were also not well discovered. Therefore, the aim of this study was to investigate the relationships among heavy metals including Pb in blood and nickel (Ni), chromium (Cr), Mn, As, Cu, and cadmium (Cd) in urine with hyperuricemia in the general population in southern Taiwan. 

## 2. Materials and Methods

### 2.1. Subject Recruitment 

We recruited participants from the general population who attended a health screening program in southern Taiwan from June 2016 to September 2018. The program was advertised in the community, and those who were willing to join the study were included. Community health examination was conducted for people over 20 years old and who have lived there for 3 years. All participants were interviewed, during which anthropometric variables (weight and height) and health surveys, including physical examinations and clinical histories, were performed and recorded by an experienced physician.

### 2.2. Collection of Demographic, Medical and Laboratory Data

Baseline variables included demographics (age and sex), medical history (diabetes mellitus [DM] and hypertension), examination findings (systolic and diastolic blood pressures [BP]) and laboratory data (fasting glucose, glycated hemoglobin [HbA1c], triglycerides, total cholesterol, hemoglobin, and uric acid). The estimated glomerular filtration rate [eGFR] was calculated using the Chronic Kidney Disease Epidemiology Collaboration estimated glomerular filtration rate equation [15]. Body mass index (BMI) was recorded as kg/m^2^. Proteinuria was examined using the dipstick method (Hema-Combistix, Bayer Diagnostics), with a result of ≥1+ being defined as positive. Blood and urine data were collected in the morning after 8 h of fasting.

### 2.3. Measurement of Heavy Metals in Blood and Urine 

Concentrations of Pb in the blood and Ni, Cr, Mn, As, Cu and Cd in the urine were measured using graphite furnace atomic absorption spectrometry (AA800v, Perkin Elmer, Waltham, MA, USA) and plasma mass spectrometry (ICP-MS, NexION 300 Series, Perkin Elmer), respectively. Details of the instrumental analysis have been described in National Institute of Environmental research. All urine heavy metals in further analyses were adjusted for urine creatinine. Daily internal and external quality control testing was performed to ensure the accuracy of the measurements. 

### 2.4. The LOQ of Each Heavy Metal

1. Blood Pb: 82 μg/L (0.384 μmol/L);
2. Urine Cd: 0.5 ppb (0.0044 μmol/L);
3. Urine Cu: 1.0 ppb (0.0157 μmol/L);
4. Urine Ni: 1.0 ppb (0.017 μmol/L);
5. Urine As: 1.0 ppb (0.01335 μmol/L);
6. Urine Cr: 0.2 ppb (0.0038 μmol/L);
7. Urine Mn: 0.5 ppb (0.0091 μmol/L).

### 2.5. Definition of Hyperuricemia

A serum uric acid level greater than 7.0 mg/dL (416.5 μmol/L) in men and 6.0 mg/dL (357 μmol/L) in women was defined as hyperuricemia [16].

### 2.6. Ethics Statement 

The study protocol was approved by the Institutional Review Board of Kaohsiung Medical University Hospital, Number: KMUHIRB-G(II)-20190011, and informed consent was obtained from each participant before entering the study.

### 2.7. Statistical Analysis

Data are shown as number (%), mean (±standard deviation), or median (25th–75th percentile) for a non-Gaussian distribution of triglycerides and heavy metals. Differences between categorical variables were checked using the chi-square test, and the independent *t*-test for continuous variables. Binary logistic regression analysis was used to evaluate associations among the heavy metals with hyperuricemia. Multivariable analysis was then performed with the significant variables in univariable analysis. For all heavy metals (in blood and urine), natural logarithm values were used. An interaction p in logistic analysis was defined as: model disease (y) = x1 + x2 + x1 × x2 + covariates, where x1 × x2 is the interaction term; y = hyperuricemia; x1 and x2 = each heavy metals. The covariates were significant variables in univariable analysis. A *p* value of less than 0.05 was considered to be significant. Statistical analyses were performed using SPSS 19.0 for Windows (IBM Corp., Armonk, NY, USA).

## 3. Results 

A total of 2447 participants (male: 977, female 1470) were included, with a mean age of 55.1 ± 13.2 years (range: 20–91 years old). The participants were classified into two groups according to whether they had (*n* = 626; 25.6%) or did not have (*n* = 1821; 74.4%) hyperuricemia.

### 3.1. Comparisons of the Characteristics between the Participants with and without Hyperuricemia

The group with hyperuricemia were older, had more men, and had a higher prevalence of hypertension and proteinuria, higher systolic and diastolic BP, BMI, uric acid, fasting glucose, HbA1c, hemoglobin, triglycerides and total cholesterol, and a lower eGFR than the group without hyperuricemia (Table 1). In addition, the group with hyperuricemia had higher concentrations of urine As and Cu, and a higher concentration of blood Pb. As for the information on drinking water, occupational exposure, and food habits, there was no significant different between the two groups.

### 3.2. Determinants of Hyperuricemia 

The results of univariable logistic regression analysis for the determinants of hyperuricemia are shown in Table 2. Old age, male sex, hypertension, high systolic and diastolic BP, BMI, fasting glucose, HbA1c, hemoglobin, triglycerides and total cholesterol, and low eGFR, and proteinuria were associated with hyperuricemia. In addition, high concentrations of urine As and Cu, and a high concentration of blood Pb were also associated with hyperuricemia.

After adjusting for significant variables in Table 2, young age (odds ratio [OR], 0.950; 95% confidence interval [CI], 0.934 to 0.965; *p* < 0.001), male sex (vs. female sex; OR, 1.623; 95% CI, 1.238 to 2.127; *p* < 0.001), high BMI (OR, 1.139; 95% CI, 1.105 to 1.173; *p* < 0.001), high hemoglobin (OR, 1.112; 95% CI, 1.019 to 1.213; *p* = 0.018), high triglycerides (OR, 4.870; 95% CI, 2.959 to 8.014; *p* < 0.001), low eGFR (OR, 0.935; 95% CI, 0.923 to 0.946; *p* < 0.001), and high urine As (log per 1 μg/g creatinine; OR, 1888; 95% CI, 1.384 to 2.574; *p* < 0.001) were significantly associated with hyperuricemia (Table 3).

### 3.3. Interactions among Heavy Metals on Hyperuricemia

We further evaluated the effects of the interactions between heave metals on hyperuricemia. In statistics, an interaction may arise when considering the relationship among variables, and describes a situation in which the effect of one causal variable on an outcome depends on the state of a second causal variable. After multivariable analysis, the interactions between Pb × Ni (*p* = 0.531), Pb × Cr (*p* = 0.598), Pb × Mn (*p* = 0.500), Pb × As (*p* = 0.213), Pb × Cu (*p* = 0.433), Pb × Cd (*p* = 0.010), Ni × Cr (*p* = 0.515), Ni × Mn (*p* = 0.846), Ni × As (*p* = 0.733), Ni × Cu (*p* = 0.002), Ni × Cd (*p* = 0.050), Cr × Mn (*p* = 0.722), Cr × As (*p* = 0.918), Cr × Cu (*p* = 0.503), Cr × Cd (*p* = 0.001), Mn × As (*p* = 0.611), Mn × Cu (*p* = 0.080), Mn × Cd (*p* = 0.705), As × Cu (*p* = 0.268), As × Cd (*p* = 0.414), and Cu × Cd (*p* = 0.319) on hyperuricemia were found. Among them, the interactions between Pb × Cd, Ni × Cu, and Cr × Cd on hyperuricemia were statistically significant (Table 4). Increasing levels of Pb and Cr yielded an increased prevalence of hyperuricemia, and the effect progressively greater for increasing Cd. Moreover, increasing levels of Ni yielded an increased prevalence of hyperuricemia, and the effect was progressively greater for increasing Cu.

### 3.4. Determinants for Hyperuricemia in Subgroup Analysis according to Age and Sex

We further performed subgroup analysis according to age and sex (Table 5). After multivariable forward analysis, no significant association between heavy metals and hyperuricemia was noted in men with age younger than 50 years. In men with age older than 50 years, high blood Pb (log per 1 μg/L; OR, 2.089; 95% CI, 1.077 to 4.053; *p* = 0.029), and high urine As (log per 1 μg/g creatinine; OR, 2.293; 95% CI, 1.264 to 4.159; *p* = 0.006) were significantly associated with hyperuricemia. In women with age younger than 50 years, high urine Cr (log per 1 μg/g creatinine; OR, 6.143; 95% CI, 1.332 to 28.318; *p* = 0.020) was significantly associated with hyperuricemia. Additionally, in women with age older than 50 years, high urine As (log per 1 μg/g creatinine; OR, 1.868; 95% CI, 1.158 to 3.013; *p* = 0.010), and high urine Cd (log per 1 μg/g creatinine OR, 1.553; 95% CI, 1.020 to 2.362; *p* = 0.040) were significantly associated with hyperuricemia.

### 3.5. Determinants for As Using Linear Regression Analysis

We further evaluated determinants of As using linear regression analysis (Table 6). Univariate analysis showed that old age, female sex, DM and hypertension history, high systolic BP, high fasting glucose, high HbA1c, high total cholesterol, low eGFR, non-occupational exposure, non-RO reverse osmosis water use, private water filling station use, drinking after boiling, eating vegetables every day, and seafood consumption were associated with high As levels. After multivariable analysis, old age (per 1 year; unstandardized coefficient β, 0.011; 95% CI, 0.008 to 0.015; *p* < 0.001), female sex (vs. male; unstandardized coefficient β, −0.059; 95% CI, −0.115 to −0.002; *p* = 0.044), high eGFR (per 1 mL/min/1.73 m^2^; unstandardized coefficient β, 0.003; 95% CI, 0 to 0.007; *p* = 0.047), and seafood consumption (unstandardized coefficient β, 0.171; 95% CI, 0.110 to 0.231; *p* < 0.001) were significantly associated with high urine As level.

## 4. Discussion

In this study, we investigated associations between seven heavy metals and hyperuricemia among 2447 Taiwanese participants, and found that a high concentration of As was associated with hyperuricemia. In addition, in subgroup analysis, we found that high blood Pb, high urine Cr, and high urine Cd were associated with hyperuricemia, and significant interactions were found between Pb and Cd, and Cr and Cd on hyperuricemia. In addition, the results showed that young age, male sex, high BMI, triglycerides and hemoglobin, and low eGFR were associated with hyperuricemia.

The first main finding is that high urine As was associated with hyperuricemia. As is considered non-essential for humans [10]. A wide variety of As compounds exists in the geosphere and biosphere [18]. These compounds were subdivided into inorganic or organic forms [10]. Inorganic As in the reduced (arsenite) and oxidized form (arsenate) are considered more toxic and carcinogenic than organic As compounds such as monomethylarsonic acid (MMA), dimethylarsinic acid (DMA), arsenolipids or arsenobetaine [18]. Inorganic As poisoning has been reported through the ingestion of drinking water and rice polluted by burning fossil fuels, agricultural pesticides and industrial waste [19]. However, these are not the only source of As in the diet, and seafood including finfish, shellfish, and seaweed has been found to be a major source of As exposure [20]. Although marine-derived foods are primarily in the form of organic compounds, which have been considered to be mostly non-toxic, their true effect and impact on human health is not yet fully understood [21]. After absorption by our gastrointestinal tract, As compounds are mainly metabolized within our livers [19]. Most of inorganic As detection is MMA or DMA, which is considered to be less toxic and more rapidly renal-excreted through eliminating protein-binding ability [19]. Most ingested As, regardless of inorganic or organic forms, is excreted via urine and can be detected by a urine test [21]. Further inorganic As and its metabolites, such as MMA and DMA, is suggested if total urine As is high [18]. The exact mechanism of As toxicity is still under debate. In a study enrolling 97 participants in Mexico to evaluate the effect of As exposure (>130 μg/L) on uric acid concentration, the authors found that a high As level was significantly related to a decrease in both serum and urine uric acid level [22]. They explained their findings by the inhibition of xanthine oxidase by As. Another study conducted in eastern India also revealed a reverse relationship between high As levels in drinking water (>100 μg/L) and uric acid level [14]. The authors demonstrated significantly decreased antioxidant profiles including catalase, soluble thiol and malondialdehyde in those exposed to a higher level of As, and concluded that the consumption of serum uric acid as an antioxidant against As induced oxidative stress leading to hypouricemia [14]. Whether the relatively high As exposure in both studies led to the different results to ours is unknown. Taking levels of As intake into consideration, in countries with low to moderate As exposure in drinking water, a large cross-sectional study with 27,152 participants from 2003~2010 in the United States showed that a relatively high As exposure, as measured in urine, was associated with an increased prevalence of hyperuricemia in men, though the effect on women was inconclusive [13]. Even after adjusting for arsenobetaine level, which is considered to be the main As metabolite in humans of organic As in seafood, the result were the same. In addition, an experiment on As-intoxicated rats showed that the rats had high levels of urea, creatinine and uric acid [23]. Furthermore, the histopathological results of kidney tissue showed renal damage, especially in the epithelial cells of proximal convoluted tubules and podocytes of the Bowman’s capsule. Hence, the apoptosis of renal tissue cells and reduced uric acid excretion may explain the association between As and hyperuricemia [23]. In our study, we found that the southern Taiwan population had relatively high urine As level compared with European populations [18]. One reason may be the high consumption of seafood in Taiwan due to the proximity to rich marine resources as with Iceland, Japan, and Korea [24]. Further speciation on inorganic As is necessary. Another finding in our study demonstrated that old age, female sex, and high eGFR were associated with high urine As, however, these factors were the opposite to hyperuricemia. This finding may imply that the relationship between As and hyperuricemia is not through these factors.

Second, we found that high blood Pb was associated with hyperuricemia in the subgroup of men with age older than 50 years. A report from the National Health and Nutrition Examination Survey (NHANES) during 2005–2008 excluding participants with age younger than 40 years showed similar result to ours but no sex difference [25]. Another report from the 2011–2018 NHANES project demonstrated that sex is a potential modifier between blood Pb and hyperuricemia, though the age stratification showed no significant difference [26]. The mechanisms underlying the effect of Pb on hyperuricemia are complex and unclear. Reports have shown that Pb may lead to renal proximal tubule damage or induce xanthine oxidase activity and may partly explain the relationship with hyperuricemia [27,28]. Additionally, significant interactions were found between Pb and Cd in hyperuricemia in our study. Concurrent exposure to both metals is very common and Pb was found to have links with Cd in several research studies [26,29,30]. A study recruiting 122 male metallurgic refinery workers found an additive effect of Pb and Cd on renal dysfunction [29]. N-acetyl-glucosaminidase (NAG) and intestinal alkaline phosphatase (IAP) were used as biomarkers of renal proximal tubular damage. This study showed statistically significant results between the excretion of NAG and IAP and Pb × Cd [29]. Impaired kidney function may further induce hyperuricemia [26].

The third finding of the study is that high urine Cr was associated with hyperuricemia in the subgroup of women with age less than 50 years. Chromium (Cr) is an important material, but also one of the most toxic heavy metal pollutants, showing a great threat to human health and the ecological environment. Cr (VI) and Cr (III) are two common forms found in the biological environment. Cr(VI) obtains a higher redox potential and a greater ability to enter the cell [31]. Hence, Cr(VI) is proved to be genotoxic and carcinogenic [32]. Cr(III) had been considered beneficial to insulin sensitivity and lipid metabolism, but recent studies concluded that these effects are highly uncertain [33,34]. Cr intoxication can lead to multiple organ damage and variable systemic reactions, ranging from allergic reaction of the respiratory tract or skin, damage of hepatocytes and renal tubular cells, and malignancy [32]. One study recruiting 186 participants in Wuhan, China showed a reverse association of urine Cr and hyperuricemia [35]. Whether urine Cr acts as a predisposing factor for hyperuricemia still needs further mechanism studies. 

The fourth finding of the study is that high urine Cd was associated with hyperuricemia in the subgroup of women with age older than 50 years. Inconsistent results have been obtained from studies examining the relationship between Cd and uric acid. In a survey of the American population, blood Cd were positively associated with increased hyperuricemia in women [36]. In Sun H’s study, blood Cd had a positive correlation in Chinese men but not in women [37]. Another report from the Korean National Health and Nutrition Examination Survey (KNHANES) 2016–2017 showed an inverse correlation between Cd and hyperuricemia in women [38]. Those discrepant results may be related to different exposure levels. The US study was found to have lower blood Cd level than the studies from China and Korea [38]. In our study, we applied the urine Cd level. Though urine Cd is considered a good biomarker of body burden, there are still limited data about the consistency of blood Cd and urine Cd [29]. Information on the sex and age difference is limited. The estrogenic effect of Cd had been considered the protective factor of hyperuricemia [39]. Whether the interaction between endogenous estrogen and Cd result in sex and age differences needs further research. In addition, significant interactions were found between Cr and Cd in hyperuricemia in our study. Both Cr and Cd were found to accumulate in our proximal tubules and may lead to nephrotoxicity [28,40], which may lead to additive effects in hyperuricemia. Further investigation is needed to elucidate this issue.

Fifth, we found that young age was associated with hyperuricemia. Due to the high prevalence of hyperuricemia in the elderly [2], old age is considered to be a risk factor for hyperuricemia, in contrast to our findings. The same conclusion has also been reported in recent studies. She et al. [41] collected data from people aged 20~79 years in eastern China between 2009 and 2019, and found that age was negatively associated with hyperuricemia in 2019, but positively correlated with hyperuricemia in 2009. A change in dietary habits may partially explain these findings with the higher consumption of red meat and alcohol, and a lower vegetable intake, and these may serve as independent risk factors for hyperuricemia [42,43]. 

Sixth, we found that male sex was associated with hyperuricemia. It is well known that men tend to have higher uric acid levels and prevalence of hyperuricemia than women [42,44]. A systematic review conducted in China from 2000 to 2014 found a sex difference in the prevalence of hyperuricemia, with a rate of 19.4% in men and 7.9% in women [44]. Data from Taiwan in 2005~2008 also showed a significant sex difference in hyperuricemia with a rate of 22.0% in men and 9.7% in women. Sex hormones may partially explain these findings. In our study, if we used the same criteria as the Nutrition and Health Surveys in Taiwan (a serum uric acid level ≥ 7.7 mg/dL [458.5 μmol/L] in men and ≥6.6 mg/dL [392.7 μmol/L] in women), the results showed that the prevalence of hyperuricemia is 21.2% in men and 12.6% in women. Mumford et al. [45] reported variations in uric acid levels during the menstrual cycle, and an inverse association between uric acid and estradiol levels. Hence, they concluded that the ovulatory cycle may have a protective effect against hyperuricemia. A relatively low uric acid level has also been reported in premenopausal women compared with postmenopausal women [42]. Estradiol has been hypothesized to increase renal uric acid clearance and reduce uric acid resorption in some studies [45,46]. Another contributor to the different prevalence of hyperuricemia between sexes may be related to dietary habit [41]. Men are prone to consuming more red meat, high-fat foods and alcohol in their daily diet than women [41], and hyperuricemia and gout have been positively correlated with the intake of red meat, seafood and alcohol, and negatively correlated with vegetable intake [47]. 

Seventh, our results showed that high BMI and high triglycerides were associated with hyperuricemia. She et al. [41] demonstrated a significant positive correlation between BMI and hyperuricemia. In addition, the association between BMI and triglycerides with hyperuricemia may be related to metabolic syndrome (MetS), of which the pathogenesis is regarded to be insulin resistance and chronic low-grade inflammation [48]. Many studies have indicated an association between hyperuricemia and MetS [49,50]. A study from South Korea found that hyperuricemia was associated with high waist circumference (WC), BP, triglycerides, and reduced high-density lipoprotein (HDL) cholesterol [50]. A Chinese study reported positive correlations between hyperuricemia with high WC, triglycerides, and BP, but not HDL-cholesterol [51], and a study from Tibet [52] reported that the risk factors for hyperuricemia were high WC, BP, and triglycerides. Differences in dietary habits, race, educational level, and occupation may explain the differences in conclusions [52]. Other studies have also demonstrated that hyperuricemia is a risk factor for high triglycerides and WC [53,54]. The pathogenesis of this bidirectional relationship between MetS and hyperuricemia is still poorly understood. Adipose tissue contains enzymes necessary for purine catabolism, such as xanthine oxidase [39], and obesity has been shown to increase the activity of xanthine oxidase and uric acid secretion from adipose tissue [55]. Uric acid may also induce oxidative stress and stimulate nicotinamide adenine dinucleotide phosphate oxidase, which is recognized as a major causative factor for obesity-related inflammation and dyslipidemia [54]. In addition, obese subjects with high levels of uric acid have been found to have lower insulin sensitivity than obese subjects with normal uric acid levels, which further implies an association between hyperuricemia and insulin resistance [56].

We also found that low eGFR was associated with hyperuricemia. Decreased renal function has been associated with hyperuricemia in previous studies [57,58,59]. Cirillo et al. reported that the prevalence of hyperuricemia was higher in people with an eGFR < 60 mL/min/1.73 m^2^ than in those with an eGFR ≥ 60 mL/min/1.73 m^2^ [58]. Oh et al. [59] also reported the tendency of a higher serum uric acid concentration in subjects with a lower eGFR. They also demonstrated that high uric acid levels were strongly correlated with an increased risk of initiating dialysis or kidney transplantation [59]. Approximately two-thirds of the uric acid in humans is excreted in urine, with one-third excreted in the gastrointestinal tract [57]. The pathophysiologic mechanism between CKD and hyperuricemia is complex and involves factors including volume status, drugs, comorbidities, degree of renal impairment, and the cause of CKD [57]. Studies on the molecular mechanisms have found that defective renal excretion of uric acid is associated with both decreased renal glomerular filtration and also dysregulation of urate transporters in proximal tubules [60]. Hence, the interplay of multiple factors in CKD patients may contribute to hyperuricemia [61].

Finally, we found the association between high hemoglobin with hyperuricemia. The occurrence of hyperuricemia with low hemoglobin is common in patients with hemolytic anemia or hematology diseases with a large tumor burden [62,63]. He et al. [64] demonstrated an association between hemoglobin and hyperuricemia. In their cohort study, participants were followed from 2011 to 2014 to verify the association between hemoglobin with MetS components and hyperuricemia, and they found that higher baseline hemoglobin was associated with low HDL-cholesterol, high triglycerides, and hyperuricemia in women, and abdominal obesity, low HDL-cholesterol, and hyperuricemia in men [64]. One hypothesis for this result may be insulin resistance. A high hemoglobin level increases blood viscosity and consequently decreases blood flow to skeletal muscles and fat tissues, further interfering with glucose and insulin delivery to essential tissues, thereby inducing an insulin-resistant state. Insulin resistance can further induce renal urate reabsorption through the stimulation of glucose transporter 9 or uric acid transporter 1 in proximal renal tubules, further contributing to an elevation in the uric acid level [65]. 

The main strength of this study is that we studied the impact of heavy metals on hyperuricemia in a large population. Nevertheless, several limitations should also be discussed. First, we only had single metal concentration measurements. Second, we could not determine causal relationships or long-term clinical outcomes due to the cross-sectional nature of the study. Long-term prospective studies with serial heavy metal measurements and measurements of changes in uric acid are needed to verify our results. Third, exposure to As was assessed using the total urine concentration. Although this method is relatively simple and therefore appropriate when required to process many samples, it cannot reflect differences in uptake and metabolism between subjects. Nevertheless, this method can reasonably reflect inorganic As exposure in clinical practice. Fourth, it was not possible to confirm the source of the heavy metals. Despite measuring serum biochemical data and blood and urine concentrations of the heavy metals, different routes of exposure may have different effects. In our study, we just have blood Pb, and urine Ni, Cr, Mn, As, Cu, and Cd. We do not have the blood and tissue heavy metal levels. In addition, all of the participants were volunteers and the percentage of females was higher, which could have led to selection bias and affected the interpretation of the results. Lastly, although the questionnaire included diet behavior, however, the contents of vegetables and the frequency and duration of seafood consumption needed to be clarified more clearly.

In conclusion, As was significantly associated with hyperuricemia, and we also found young age, male sex, high BMI, high hemoglobin, high triglycerides, and low eGFR were significantly associated with hyperuricemia. Our results show that As may have an impact on the development of hyperuricemia, and offer clinical evidence of the metabolic effect of heavy metal exposure. In addition, the interactions between Pb × Cd, Ni × Cu, and Cr × Cd in hyperuricemia were statistically significant. Our results show that high urine As is associated with hyperuricemia, and some interactions of heavy metals on hyperuricemia are noted.

## Figures and Tables

**Table 1:** Comparison of clinical characteristics among participants according to hyperuricemia in study participants.

Characteristics	Hyperuricemia (−) (*n* = 1821)	Hyperuricemia (+) (*n* = 626)	*p*
Age (years)	54.2 ± 12.8	57.7 ± 13.9	<0.001
Male (%)	35.6	52.6	<0.001
DM (%)	10.2	11.2	0.495
Hypertension (%)	22.1	34.8	<0.001
Systolic BP (mmHg)	130.6 ± 19.8	136.4 ± 19.2	<0.001
Diastolic BP (mmHg)	76.8 ± 11.5	79.8 ± 11.8	<0.001
BMI (kg/m^2^)	24.4 ± 3.8	26.8 ± 4.0	<0.001
Laboratory parameters			
Uric acid (mg/dL)	5.0 ± 1.0	7.7 ± 1.2	<0.001
Fasting glucose (mg/dL)	99.2 ± 27.3	101.9 ± 27.4	0.036
HbA1c (%)	5.8 ± 1.0	5.9 ± 0.9	0.009
Hemoglobin (g/dL)	13.9 ± 1.6	14.4 ± 1.6	<0.001
Triglyceride (mg/dL)	96 (68–137)	131 (92.75–186.25)	<0.001
Total cholesterol (mg/dL)	198.6 ± 37.0	202.6 ± 38.7	0.026
eGFR (mL/min/1.73 m^2^)	91.3 ± 14.4	82.5 ± 19.5	<0.001
Proteinuria (%)	8.8	14.7	<0.001
Heavy metals			
Blood			
Pb (μg/L)	15 (10–21)	17 (11–24)	<0.001
Urine			
Ni (μg/g creatinine)	2.5 (1.6–3.9)	2.3 (1.5–3.4)	0.343
Cr (μg/g creatinine)	0.1 (0.1, 0.1)	0.1 (0.1, 0.1)	0.179
Mn (μg/g creatinine)	1.7 (0.9–3.0)	1.8 (0.9–2.9)	0.463
As (μg/g creatinine)	75.1 (42.4–134.5)	87.8 (53.4–156.6)	<0.001
Cu (μg/dg creatinine)	1.4 (1.0–1.9)	1.5 (1.1–2.0)	0.004
Cd (μg/g creatinine)	0.9 (0.5–1.4)	0.8 (0.5–1.3)	0.330
Occupational exposure ^a^ (%)	21.3	20.2	0.638
Main source of drinking water			
Commercially available mineral water (%)	18.2	21.5	0.151
RO reverse osmosis water (%)	38.7	34.4	0.117
Groundwater (%)	0.5	0.5	0.968
Tap water (%)	15.3	15.4	0.972
Private water filling station (%)	47.6	51.5	0.173
Drinking after boiling (%)	91.2	89.4	0.278
Eat vegetables every day (%)	66.2	62.1	0.132
Seafood consumption in recent 3 days (%)	73.1	71.8	0.750

Abbreviations. DM, diabetes mellitus; BP, blood pressure; BMI, body mass index; HbA1c, glycated hemoglobin; eGFR, estimated glomerular filtration rate; Pb, lead; Ni, nickel; Cr, chromium; Mn, manganese; As, arsenic; Cu, copper; Cd, cadmium. ^a^ Ever vs. never exposed to fiberglass, silica, diesel exhaust, wood dust, arsenic, asbestos, welding fumes, other fumes, coke oven emissions, soot, acrylic, beryllium, or radon at work. Reference values (RV95) for metals (μg/L): blood Pb: 33 (31–36), urine Ni: 4.4 (3.8–5.0), urine Cr: 0.22 (0.20–0.25), urine Mn: 4 (3.6–4.5), urine As:27 (20–34), urine Cu: 25 (22–28) and urine Cd: 1.3 (1.1–1.5) [17].

**Table 2:** Determinants for hyperuricemia using univariable logistic regression analysis.

Variables	Univariable (Hyperuricemia)	
	Odds Ratio (95% CI)	*p*
Age (per 1 year)	1.021 (1.013–1.028)	<0.001
Male (vs. female)	2.005 (1.668–2.410)	<0.001
DM	1.107 (0.827–1.481)	0.495
Hypertension	1.886 (1.547–2.300)	<0.001
Systolic BP (per 1 mmHg)	1.015 (1.010–1.019)	<0.001
Diastolic BP (per 1 mmHg)	1.022 (1.014–1.030)	<0.001
BMI (per 1 kg/m^2^)	1.167 (1.139–1.196)	<0.001
Laboratory parameters		
Fasting glucose (per 1 mg/dL)	1.003 (1.000–1.006)	0.037
HbA1c (per 1%)	1.126 (1.029–1.233)	0.010
Hemoglobin (per 1 g/dL)	1.212 (1.143–1.286)	<0.001
Triglyceride (log per 1 mg/dL)	10.929 (7.285–16.395)	<0.001
Total cholesterol (per 1 mg/dL)	1.003 (1.000–1.005)	0.022
eGFR (per 1 mL/min/1.73 m^2^)	0.969 (0.964–0.974)	<0.001
Proteinuria	1.776 (1.351–2.336)	<0.001
Blood		
Pb (log per 1 μg/L)	2.179 (1.562–3.039)	<0.001
Urine		
Ni (log per 1 μg/g creatinine)	0.930 (0.801–1.080)	0.343
Cr (log per 1 μg/g creatinine)	1.483 (0.878–2.504)	0.140
Mn (log per 1 μg/g creatinine)	0.939 (0.793–1.111)	0.463
As (log per 1 μg/g creatinine)	1.698 (1.322–2.181)	<0.001
Cu (log per 0.1 μg/g creatinine)	1.676 (1.161–3.421)	0.006
Cd (log per 1 μg/g creatinine)	0.902 (0.732–1.110)	0.329
Occupational exposure ^a^	0.935 (0.706–1.237)	0.638
Main source of drinking water		
Commercially available mineral water	1.225 (0.928–1.618)	0.152
RO reverse osmosis water	0.829 (0.655–1.048)	0.117
Groundwater	0.967 (0.194–4.182)	0.968
Tap water	1.006 (0.736–1.373)	0.972
Private water filling station	1.169 (0.934–1.463)	0.173
Drinking after boiling	0815 (0.562–1.180)	0.279
Eat vegetables every day	0.837 (0.664–1.055)	0.132
Seafood consumption in recent 3 days	0.937 (0.628–1.399)	0.751

Values expressed as odds ratio and 95% confidence interval (CI). Abbreviations are the same as in Table 1. ^a^ Ever vs. never exposed to fiberglass, silica, diesel exhaust, wood dust, arsenic, asbestos, welding fumes, other fumes, coke oven emissions, soot, acrylic, beryllium, or radon at work.

**Table 3:** Determinants for hyperuricemia using multivariable logistic regression analysis.

Variables	Multivariable (Hyperuricemia)	
	Odds Ratio (95% CI)	*p*
Age (per 1 year)	0.950 (0.934–0.965)	<0.001
Male (vs. female)	1.623 (1.238–2.127)	<0.001
Hypertension	1.045 (0.807–1.355)	0.737
Systolic BP (per 1 mmHg)	1.000 (0.992–1.008)	0.997
Diastolic BP (per 1 mmHg)	1.003 (0.991–1.015)	0.642
BMI (per 1 kg/m^2^)	1.139 (1.105–1.173)	<0.001
Laboratory parameters		
Fasting glucose (per 1 mg/dL)	0.994 (0.987–1.001)	0.109
HbA1c (per 1%)	0.946 (0.762–1.175)	0.616
Hemoglobin (per 1 g/dL)	1.112 (1.019–1.213)	0.018
Triglyceride (log per 1 mg/dL)	4.870 (2.959–8.014)	<0.001
Total cholesterol (per 1 mg/dL)	1.000 (0.997–1.003)	0.834
eGFR (per 1 mL/min/1.73 m^2^)	0.935 (0.923–0.946)	<0.001
Proteinuria	1.051 (0.721–1.528)	0.795
Blood		
Pb (log per 1 μg/L)	1.221 (0.820–1.817)	0.327
Urine		
As (log per 1 μg/g creatinine)	1.888 (1.384–2.574)	<0.001
Cu (log per 0.1 μg/dg creatinine)	0.705 (0.420–1.184)	0.187

Values expressed as odds ratio and 95% confidence interval (CI). Abbreviations are the same as in Table 1. Covariates in the multivariable model included age, sex, hypertension, BMI, systolic and diastolic BPs, fasting glucose, HbA1c, log triglyceride, total cholesterol, eGFR, proteinuria, Pb, As and Cu (significant variables in Table 2).

**Table 4:** The interaction between heavy metals on hyperuricemia.

Heavy Metals	Interaction	
	Odds Ratio (95% CI)	*p*
Pb	1.569 (1.026–2.399)	0.038
Cd	0.260 (0.089–0.759)	0.014
Pb× Cd	3.152 (1.323–7.509)	0.010
Ni	0.888 (0.723–1.090)	0.255
Cu	0.927 (0.543–1.583)	0.781
Ni× Cu	0.387 (0.214–0.701)	0.002
Cr	1.813 (0.980–3.354)	0.058
Cd	8.576 (2.461–29.881)	0.001
Cr× Cd	9.090 (2.550–32.402)	0.001

Values expressed as odds ratio and 95% confidence interval (CI). Abbreviations are the same as in Table 1. Covariates in the multivariable model included age, sex, hypertension, BMI, systolic and diastolic BPs, fasting glucose, HbA1c, log triglyceride, total cholesterol, and eGFR (significant variables in Table 2).

**Table 5:** Determinants for hyperuricemia in subgroup analysis according to age and sex.

Heavy Metals	Multivariable (Forward)	
	Odds Ratio (95% CI)	*p*
Men with age older than 50 years		
Blood Pb (log per 1 μg/L)	2.089 (1.077–4.053)	0.029
Urine As (log per 1 μg/g creatinine)	2.293 (1.264–4.159)	0.006
Women with age younger than 50 years		
Urine Cr (log per 1 μg/g creatinine)	6.143 (1.332–28.318)	0.020
Women with age older than 50 years		
Urine As (log per 1 μg/g creatinine)	1.868 (1.158–3.013)	0.010
Urine Cd (log per 1 μg/g creatinine)	1.553 (1.020–2.362)	0.040

Values expressed as odds ratio and 95% confidence interval (CI). Abbreviations are the same as in Table 1. Covariates in the multivariable model included age, sex, hypertension, BMI, systolic and diastolic BPs, fasting glucose, HbA1c, log triglyceride, total cholesterol, eGFR, and each heavy metal (significant variables in Table 2).

**Table 6:** Determinants for urine As using linear regression analysis.

Variables	Univariable		Multivariable	
	Unstandardized Coefficient β (95% CI)	*p*	Unstandardized Coefficient β (95% CI)	*p*
Age (per 1 year)	0.009 (0.008, 0.010)	<0.001	0.011 (0.008, 0.015)	<0.001
Male (vs. female)	−0.056 (−0.086, −0.026)	<0.001	−0.059 (−0.115, −0.002)	0.044
DM	0.077 (0.029, 0.125)	0.002	−0.007 (−0.143, 0.129)	0.921
Hypertension	0.104 (0.071, 0.138)	0.002	0.007 (−0.071, 0.085)	0.858
Systolic BP (per 1 mmHg)	0.003 (0.002, 0.004)	<0.001	0 (−0.002, 0.002)	0.923
Diastolic BP (per 1 mmHg)	0 (−0.001, 0.001)	0.758	-	-
BMI (per 1 kg/m^2^)	0.003 (0, 0.007)	0.075	-	-
Laboratory parameters				
Fasting glucose (per 1 mg/dL)	0.001 (0.001, 0.002)	<0.001	−0.001 (−0.003, 0.001)	0.350
HbA1c (per 1%)	0.045 (0.029, 0.060)	<0.001	0.017 (−0.043, 0.076)	0.577
Hemoglobin (per 1 g/dL)	−0.004 (−0.013, 0.005)	0.352	-	-
Triglyceride (log per 1 mg/dL)	−0.045 (−0.107, 0.017)	0.153	-	-
Total cholesterol (per 1 mg/dL)	0.001 (0, 0.001)	0.003	0 (−0.001, 0)	0.357
eGFR (per 1 mL/min/1.73 m^2^)	−0.005 (−0.006, −0.004)	<0.001	0.003 (0, 0.007)	0.047
Proteinuria	0.007 (−0.041, 0.056)	0.760	-	−
Occupational exposure ^a^	−0.117 (−0.162, −0.073)	<0.001	0.008 (−0.059, 0.075)	0.812
Main source of drinking water				
Commercially available mineral water	−0.044 (−0.090, −0.002)	0.063	-	-
RO reverse osmosis water	−0.089 (−0.126, −0.051)	<0.001	−0.030 (−0.097, 0.036)	0.369
Groundwater	0.117 (−0.140, 0.373)	0.373	-	-
Tap water	−0.006 (−0.044, 0.057)	0.802	-	-
Private water filling station	0.070 (0.034, 0.106)	<0.001	0.039 (−0.027, 0.105)	0.248
Drinking after boiling	0.073 (0.011, 0.135)	0.022	−0.006 (−0.090, 0.079)	0.897
Eat vegetables every day	0.050 (0.013, 0.088)	0.009	0.042 (−0.013, 0.097)	0.130
Seafood consumption in recent 3 days	0.143 (0.081, 0.205)	<0.001	0.171 (0.110, 0.231)	<0.001

Values expressed as unstandardized coefficient β and 95% confidence interval (CI). Abbreviations are the same as in Table 1. ^a^ Ever vs. never exposed to fiberglass, silica, diesel exhaust, wood dust, arsenic, asbestos, welding fumes, other fumes, coke oven emissions, soot, acrylic, beryllium, or radon at work.

## Data Availability

Data may be available upon request to interested researchers. Please send data requests to: Szu-Chia Chen, Division of Nephrology, Department of Internal Medicine, Kaohsiung Medical University Hospital, Kaohsiung Medical University.
